# Direct cerebello-striatal loop in dystonia as a possible new target for deep brain stimulation: A revised view of subcortical pathways involved

**DOI:** 10.3389/fneur.2022.912818

**Published:** 2022-08-24

**Authors:** Ryuji Kaji

**Affiliations:** ^1^Department of Clinical Neuroscience, Tokushima University, Tokushima, Japan; ^2^National Hospital Organization Utano Hospital, Kyoto, Japan

**Keywords:** dystonia, tonic vibration reflex, subcortical pathway, deep brain stimulation, cerebellum, target

## Abstract

Dystonia is the second most common movement disorder next to tremor, but its pathophysiology remains unsettled. Its therapeutic measures include anti-cholingerics and other medications, in addition to botulinum neurotoxin injections, and stereotaxic surgery including deep brain stimulation (DBS), but there still remain a number of patients resistant to the therapy. Evidence has been accumulating suggesting that basal ganglia in association with the cerebellum are playing a pivotal role in pathogenesis. Clinical observations such as sensory tricks and the effects of muscle afferent stimulation and blockage suggest the conflict between the cortical voluntary motor plan and the subcortical motor program or *motor subroutine* controlling the intended action semi-automatically. In this review, the current understanding of the possible pathways or loops involved in dystonia is presented, and we review promising new targets for Deep Brain Stimulation (DBS) including the cerebellum.

## Introduction

Dystonia is a syndrome in which sustained or repetitive muscle contractions result in twisting and repetitive movements or abnormal fixed postures (1). Usually, those muscle activities are uncontrollable by the subject and classified as an involuntary movement (2). Focal dystonias such as writer's cramp usually affect writing, but not other tasks. Abnormal contractions of muscles start as soon as the subject intend to write, and occur both in agonists (e.g., wrist flexor) and antagonists (e.g., wrist extensor), resulting in freezing of the joint (*co-contraction*), or reciprocally in those muscles (dystonic tremor), in an unintended manner. Distant muscles unnecessary for the task may also be activated (*motor overflow*). There is a discrepancy between the intended motor plan and the resultant movements.

## Task specificity and sensory trick

Tasks affected by these focal dystonias are usually performed automatically; writing, playing musical instruments, using a putter in golfing, and so on. The modalities of these tasks are obtained by intensive training with or without psychological stress. Cervical dystonia and blepharospasm are the most prevalent dystonias, and they can be regarded as derangement of head control and blinking, which are acquired after birth (3). These task specificities may be lost as the disease progresses, and the symptoms may spread over other parts of the body. Thus, dystonia is a disorder of motor programs or *subroutines* to perform semi-automatic or fully learned motor acts (3). This of course is entirely different from “supervised” motor learning process of fine control of the limbs, such as pulling a thread through a pin needle hole, which usually requires the subject's highest attention. The latter is presumably controlled by the cortico-cerebellar system (4). The pathway activated in fully learned motor tasks affected in dystonia is classified as “reinforced” learning circuit through the basal ganglia through dopaminergic system (4). As the motor skill and efficiency improve, the shift from supervised to reinforced learning with more involvement of the basal ganglia occurs (5).

Another peculiar characteristic of dystonia is a phenomenon of symptomatic improvements with the aid of sensory input to a particular part of the body when performing the task (*sensory trick*) (6, 7). For instance, touching a part of the face with the subject's hand may straighten the neck in cervical dystonia. This raised a question of stimulating the muscle spindle afferents by a tonic vibration reflex (TVR) maneuver, or blocking them by intramuscular injection of diluted lidocaine, which is known to block gamma-efferents to the spindles selectively in dystonia patients (8). Interestingly the dystonic movements were reproduced by TVR, and abated by muscle afferent block [see videos attached to (8)]. The reflex is spared in extensive cortical lesions in stroke (9). It is therefore concluded that the neural pathways involved in dystonia is mainly subcortical, and there seems to be a conflict between cortical voluntary motor command and the abnormal output from the subcortical structures. Compensatory mechanisms may be possible at the cortical level, as exemplified by the phenomenon of sensory trick, whereby the subject can find a compromise between the two systems.

Based upon the above study of TVR and muscle afferent block, it was proposed that dystonia is a sensory disorder (10). A number of the subsequent studies have explored the abnormalities of the tactile sensory discrimination of the affected or unaffected limbs in dystonia, deciphering the primary somatosensory cortex as the site of primary lesion, but they failed to show conclusive evidence whether the changes are primary or not (11–13). Temporal tactile discrimination, which mirrors the function of somatosensory cortex, was found abnormal even in psychogenic dystonia (14).

## Pathophsiology of dystonia

The exact pathology involved in dystonia is still an open question. Traditionally it is regarded as a basal ganglia disorder, since hemi-dystonia is a consequence of contralateral basal ganglia lesions (15), and remarkable histopathological loss of striosome compartment in the striatum is found in dystonic phase of X-linked dystonia-parkinsonism (XDP) (16, 17). Accumulating evidence on the other hand suggests the cerebellum in association with the striatum causing dystonia (18). An autopsy study in a hereditary case of pure dystonia demonstrated exclusive cerebellar atrophy and loss of Purkinje cells in the anterior lobe (19). Dystonia is often a presenting symptom in spinocerebellar atrophies such as SCA6 (20, 21). Of course, secondary involvement of these structures is possible, despite the lack of visibility of the primary lesion. Rare autopsy cases of primary cervical dystonia revealed patchy loss of Purkinje cells, also pointing to the cerebellum as a site of lesion (22), whereas most of the cases of idiopathic dystonia lack such pathology, indicating abnormal synaptic plasticity being the plausible cause.

## Classical model of basal ganglia circuit

**Figure 1:**
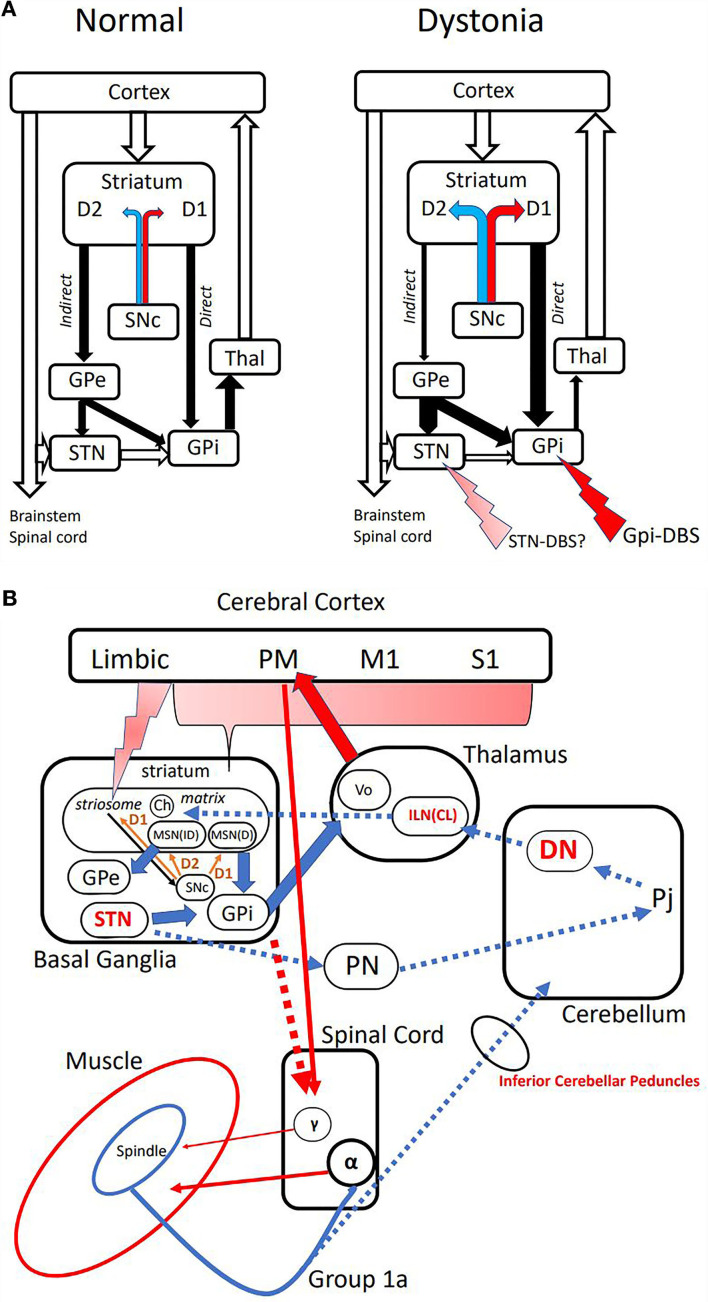
**(A)** Classical model of basal ganglia [Alexander and Crutcher (23)]. Left: Normal condition depicting “excitatory” direct and “inhibitory” indirect pathways. The majority is the inhibitory indirect path, which constitutes “surround inhibition” around the direct path activity of allowing activation of selected muscles. Open arrows are excitatory glutamatergic, and closed arrows are inhibitory GABAergic projections. Dopaminergic projections terminate on medium spiny neurons (MSNs) with excitatory D1 receptor on the direct, and inhibitory D2 receptor on the indirect pathways. Right: Suggested model in dystonia. Relative excess of dopamine from SNc produces direct pathway predominance, and disintegrates surround inhibition. Putative sites of action of DBS are shown with red arrows. SNc, Substantia Nigra pars compacta; GPe, Globus Pallidus externus; STN, Subthalamic Nucleus; GPi, Globus Pallidus internus; Thal, Thalamus. **(B)** Pathways proposed in the pathogenesis of dystonia and TVR-induced dystonic movements. Cerebral Cortex: PM premotor area, M1 primary motor area, S1 primary somatosensory area. Basal Ganglia: Ch cholinergic interneurons, MSN(D) medium spiny neuron in direct pathway, MSN(ID) medium spiny neuron in indirect pathway. Thalamus: Vo thalamic ventral-oralis complex, ILN(CL) intralaminar nuclei in primates or centro-lateral nucleus in rodents. Cerebellum: DN dentate nucleus, Pj Purkinje cells. PN, pontine nuclei. Spinal Cord: **α**-α motoneuron, **γ**-γ motoneuron. Broken arrows are putative pathway mediating TVR-induced movements, and possible targets are shown in red.

More than 3 decades ago, Alexander and Crutcher presented a “push-pull” model of basal ganglia circuit which nicely explains hypokinetic and hyperkinetic disorders such as Parkinson's disease and dystonia (23). This model is still useful in understanding dopamine excess causing hyperkinetic states such as dopa-induced dyskinesia and dystonia. There are *direct* or cortico-striato-GPi pathways that exerts excitatory feedback to the cortex (mainly premotor area or PM), and indirect or cortico-striato-GPe(STN)-GPi pathway which feeds back inhibitory background as a surround inhibition (Figure 1A). The net result would be focusing the muscles to be activated for performing tasks. In dopamine deficiency such as in Parkinson's disease, there exists more indirect pathway activity because of the lack of dopamine disinhibits medium spiny neurons (MSNs) in the striatum through D2 receptors. MSNs in the direct pathway on the contrary are inhibited through the D1 receptor. The paucity and slowness of movements (*akinesia* and *bradykinesia*) are the consequences. Dystonia is explained by the excess of dopamine, which favors a direct pathway, which activates muscles unnecessary for performing tasks, as in the case of co-contractions.

## Revised model of the basal ganglia

If the dopamine excess is the cause of dystonia, there remain conditions that are unexplained by the classical model; tardive dystonia and dopa-responsive dystonia. As most of the anti-psychotic drugs are termed atypical, causing less tardive syndrome, there still remain a large number of psychiatric patients who suffer from dystonia while using dopaminergic antagonists (24). Of course, many of them start their symptoms after reducing the dose of drugs or even stopping them, which can be explained by the super-sensitivity of the receptors after removing the blockage. Another question is dopa-responsive dystonia, which dramatically benefits from dopa administrations.

The only known pathological finding of dystonia is probably that of X-linked dystonia-parkinsonism (XDP) (16). XDP is a biphasic disease, endemic in the Panai Island of the Philippines, starting with dystonia, which gradually proceeds to Parkinsonism. At the dystonic stage, it typically presents with focal dystonia involving the jaw then generalized to the trunk and the lower limbs. MRI finding of the brain at this stage shows spot-like lesions in the putamen (16, 17, 25). Those afflicted by this condition tend to die by suicide, and autopsy findings of pure dystonia are a reality (16). The striatum consists of two compartments immunohistochemically; *striosome* and *matrix*. The lesions seen on MRI turned out to be exclusively striosome, since all the remaining MSNs are of matrix. Striosome has inputs from the limbic cortex and is related to reward-oriented control of movements (26, 27). There are dopaminergic projections from Substantia Nigra pars compacta (SNc) to the matrix as well as their axonal collaterals to striosomal MSNs with excitatory D1 receptors, which in turn send its GABAergic inhibitory axons back to nigral dopaminergic neurons, thus forming a feed-back control loop of dopamine release to the striatum (Figure 1B) (16, 25, 28). If striosomal MSNs are depleted, the control of dopamine content would be deranged, so that relative dopamine excess might result in direct pathway preponderance, causing dystonia. In a model of dopa-responsive dystonia, it was found that tyrosine hydroxylase (TH) content was more depleted in the striosome, accounting for the imbalance between the compartments causing dystonia (29). Compartmental imbalance has also been implicated in tardive dystonia (24).

## Direct connections between the basal ganglia and the cerebellum

Dystonic movements are subconscious since any volitional efforts to correct them are not possible except for the *sensory trick* maneuver. This is in contrast with *tic*, where sensory symptoms to urge movements are usually perceived by the subject, who could volitionally control the movements albeit momentarily. As mentioned above, activation of muscle afferents by high-frequency vibratory stimulation (TVR) could reproduce the dystonic movements, apart from the subject's intention. Patients with pure cerebellar pathology could present with dystonia. It is therefore reasonable to assume a subcortical circuit mediating TVR-induced dystonic movements (9), possibly including the cerebellum, where muscle spindle afferents are utilized as kinesthetic control (30).

Contributions from the basal ganglia and the cerebellum to the genesis of dystonia have been discussed in association with the cerebral cortex as separate loops (Table 1) (17, 18, 31). There has been however ample anatomical evidence showing direct di-synaptic connections between the cerebellum and the striatum or the subthalamic nucleus (32–35). Using the rabies virus as a probe, Hoshi et al. found that the striatum has a di-synaptic input from the dentate through the intralaminar nucleus (CL) of the thalamus (32). Conversely, the subthalamic nucleus (STN) was shown to have di-synaptic output to the cerebellar cortex *via* pontine nuclei (34). Chen et al. confirmed short-latency (~10 ms) cerebellar modulation of the basal ganglia between the dentate nucleus and the striatum in normal and dystonia model mouse (36). More importantly, they found that high-frequency stimulation of the cortex alone produced long-term depression (LTD), while the concurrent stimulation of the cerebral cortex and the cerebellum produced long-term potentiation (LTP) at the cortico-striatal synapses, providing the direct evidence of cerebellar inputs to the striatum modulating its neuroplasticity. They also explored the pathway in a mouse model of dystonia and found that the aberrant high-frequency inputs from the cerebellum set the cortico-striatal synapse to favoring abnormal LTP. Severing the link from the cerebellum by silencing the thalamic nuclei abolished the dystonic symptoms. These findings are relevant to the pathogenesis and treatment of dystonia since abnormal LTP or its depotentiation at the cortico-striatal synapses have been shown in a prototypic mouse model of dopa-induced dyskinesia (37) and humans with DYT1 dystonia (38, 39). The termination of the cerebelo-thalamo-striatal projection seems to be cholinergic interneurons, which are located at the border of striosome and matrix compartments, and upregulate dopamine release in the striatum *via* both nicotinic and muscarinic receptors (40, 41). It follows that dystonia can be treated with anti-cholinergics, such as trihexyphenidyl (42), and is aggravated by nicotine (43).

**Table 1:** Key references in exploring pathophysiology of dystonia.

**Anatomical basis**
Marsden CD, Obeso JA, Zarranz JJ, Lang AE. The anatomical basis of symptomatic hemidystonia. *Brain*. (1985) 108(Pt 2):463–83. 10.1093/brain/108.2.463
**Model of basal ganglia circuits**
Alexander GE, Crutcher MD. Functional architecture of basal ganglia circuits: neural substrates of parallel processing. *Trends Neurosci*. (1990) 13:266–71. 10.1016/0166-2236(90)90107-L
**Sensory aspects in dystonia**
Kaji R, Rothwell JC, Katayama M, Ikeda T, Kubori T, Kohara N, et al. Tonic vibration reflex and muscle afferent block in writer's cramp. *Ann Neurol*. (1995) 38:155–62. 10.1002/ana.410380206
Hallett M. Is dystonia a sensory disorder? *Ann Neurol*. (1995) 38:139–40. 10.1002/ana.410380203
**Somatosensory cortex**
Bara-Jimenez W, Catalan MJ, Hallett M, Gerloff C. Abnormal somatosensory homunculus in dystonia of the hand. *Ann Neurol*. (1998) 44:828–31. 10.1002/ana.410440520
**Deep brain stimulation in dystonia**
Vercueil L, Pollak P, Fraix V, Caputo E, Moro E, Benazzouz A, et al. Deep brain stimulation in the treatment of severe dystonia. *J Neurol*. (2001) 248:695–700. 10.1007/s004150170116 **Dystonia pathology in X-linked dystonia-Parkinsonism**
Goto S, Lee LV, Munoz EL, Tooyama I, Tamiya G, Makino S, et al. Functional anatomy of the basal ganglia in X-linked recessive dystonia-Parkinsonism. *Ann Neurol*. (2005) 58:7–17. 10.1002/ana.20513
**Cerebellar involvement**
Neychev VK, Fan X, Mitev VI, Hess EJ, Jinnah HA. The basal ganglia and cerebellum interact in the expression of dystonic movement. *Brain*. (2008) 131:2499–509. 10.1093/brain/awn168
**Direct cerebello-striatal projection in dystonia model**
Chen CH, Fremont R, Arteaga-Bracho EE, Khodakhah K. Short latency cerebellar modulation of the basal ganglia. *Nat Neurosci*. (2014) 17:1767–75. 10.1038/nn.3868
**Subcortical loop**
Kaji R, Bhatia K, Graybiel AM. Pathogenesis of dystonia: is it of cerebellar or basal ganglia origin? *J Neurol Neurosurg Psychiatry*. (2018) 89:488–92. 10.1136/jnnp-2017-316250

## New targets for deep brain stimulation

Targets for deep brain stimulation (DBS) for dystonia have been evolving around internal Globus Pallidum (GPi) and the subthalamic nucleus (STN). The rationale for GPi-DBS is to inhibit the direct pathway through increasing the GABAergic output from GPi to Vo thalamus, which in turn decreases the thalamo-cortical excitatory projections to the premotor area, which is known to be hyper-excitable in dystonia (44). However, STN-DBS in dystonia is less clear. In Parkinson's disease, it is expected to reduce the hyperactive indirect pathway, since STN is located in the indirect pathway, where stimulation is supposed to apply presynaptic inhibition to STN. The stimulation could reduce the ratio of indirect/direct pathways, thus improving the akinesia and bradykinesia on its own and, as a consequence, reduce the doses of anti-Parkinsonian medications. Drug-induced dystonia or dyskinesia can be improved through decreased medication.

It is also known that STN-DBS is equally effective in treating idiopathic and hereditary dystonias compared to GPi-DBS (45–47), although the stimulation parameters could differ from those in Parkinson's disease (48). The rationale for this target is not clearly explained, asin dystonia direct pathway predominance must be met with increasing indirect pathway including STN. It is therefore conceivable that stimulation at STN in dystonia could be excitatory to STN neurons in contrast to the inhibitory nature in Parkinson's. There is also a possibility that STN modulation could affect the direct di-synaptic STN-cerebellar pathway (34).

As discussed, the dentate nucleus or thalamic intralaminar nuclei are promising new targets in the light of their capability of affecting striatal neuroplasticity. In fact, dentate DBS has been reported with preliminary results (49–51). Intralaminar nuclei of the thalamus are also considered as a candidate (52). As in muscle afferent block using diluted lidocaine, the input to the cerebellum through the inferior cerebellar peduncles may be functionally manipulated, although direct evidence is lacking. The stimulation parameters and the precise location in these targets are yet to be determined.

## Conclusion

The pathways mediating abnormal motor outputs in dystonia is still undetermined. The classical “push-pull” model of Alexander-Crutcher is still useful, but many revisions must be made considering new clinical and therapeutic features of dystonia or its animal models. More clinical and animal studies searching new and unexplored targets including subcortical cerebello-thalamo-striatal or STN-ponto-cerebellar pathways are needed for better understanding and optimal treatment of dystonia.

## Author contributions

RK wrote and reviewed the manuscript.

## Funding

This work was funded by an unlimited research grant from Kyowa-Kirin Co. Ltd., Japan (KKCS2020531033) which also covered the publication fee. The funder was not involved in the study design, collection, analysis, interpretation of data, the writing of this article or the decision to submit it for publication.

## Conflict of interest

The author declares that the research was conducted in the absence of any commercial or financial relationships that could be construed as a potential conflict of interest.

## Publisher's note

All claims expressed in this article are solely those of the authors and do not necessarily represent those of their affiliated organizations, or those of the publisher, the editors and the reviewers. Any product that may be evaluated in this article, or claim that may be made by its manufacturer, is not guaranteed or endorsed by the publisher.
